# Differences in predicting athletic burnout and in moderating its relationship with life satisfaction in competitive and leisure athletes

**DOI:** 10.1038/s41598-024-74908-1

**Published:** 2024-10-22

**Authors:** Karolina Grebner, Alena Michel-Kröhler, Tabea Werner, Stefan Berti, Michèle Wessa

**Affiliations:** 1 Department of Clinical Psychology and Neuropsychology, Institute for Psychology, Johannes Gutenberg-University Mainz, Wallstrasse 3, 55122 Mainz, Germany; 2 Leibniz-Institute for Resilience Research, AG Wessa, Mainz, Germany; 3 Division Cancer Survivorship and Psychological Resilience, German Cancer Research Centre, Heidelberg, Germany; 4 Department of Neuropsychology and Psychological Resilience Research, Central Institute of Mental Health, Mannheim, Germany

**Keywords:** Integrated model of athletic burnout, Stress, Entrapment, Coping resources, Human behaviour, Risk factors

## Abstract

The effects of athletic burnout on life satisfaction vary greatly between individuals, but few studies have examined influencing factors, such as coping mechanisms, that explain these differences. While athletes’ performance levels seem to influence the development of burnout symptoms, there is a lack of studies examining different performance levels separately. The present study therefore investigated the predictors of athletic burnout in competitive and leisure athletes separately, as well as possible moderators influencing the relationship between burnout and life satisfaction in these groups. A cross-sectional online study with sport-specific and general questionnaires was conducted. Latent (e.g., resilience) and manifest variables (e.g., stress) were included as predictors of athletic burnout in two structural equation models (competitive: robust RMSEA = 0.065, robust CFI = 0.946; leisure: robust RMSEA = 0.067, robust CFI = 0.937) with data from 422 athletes (*M*_age_ = 23.65; range = 16–67; 43% female, 57% male). Additionally, moderation analyses with coping mechanisms as moderators between burnout and life satisfaction were conducted. Results show that predictors of athletic burnout differ between performance levels. Furthermore, there is a significant moderation effect (*p* < 0.01) for positive self-concept in competitive sports. Approaches for future research and the development of target group-specific interventions are discussed.

## Introduction

Although the public perceives athletes as particularly healthy and capable individuals, mental health problems of competing athletes have recently become more and more salient through their public disclosures (e.g., Simone Biles, Naomi Osaka, Michael Phelps). Indeed, up to 11% of athletes are affected by athletic burnout at some point^1^, and a recent cross-temporal meta-analysis has shown that burnout symptoms in athletes have significantly increased over the past 20 years^2^. This finding is even more concerning as athletic burnout is associated with an increased risk of developing a mental disorder (e.g., depression) and with more general negative consequences, such as lower life satisfaction^3^. The effects of athletic burnout on life satisfaction, however, vary greatly between individuals. Only a few studies, though, have examined psychological or sport-specific factors that explain individual differences in the mental health consequences of burnout (e.g., Wang et al.^4^), whereas more studies investigated predictors of athletic burnout itself. One of such predictors is the athletic performance level, which appears to play a role in the development of athletic burnout symptoms^5^. Yet, studies comparing (1) predictors of athletic burnout and (2) factors influencing the relationship between athletic burnout and life satisfaction between different athletic performance levels are lacking to date.

Athletic burnout has been defined as a multidimensional syndrome consisting of three dimensions: (1) physical and emotional exhaustion, which is linked to intensive training and competitions and relates to fatigue, (2) sport devaluation, which is associated with negative attitudes towards sport and engagement in it, and (3) reduced sense of accomplishment, reflecting negative evaluations of oneself and the own performance quality^6^. Several theoretical models conceptualize athletic burnout as a reaction to stress^7^. In addition to general life-events and daily hassles, high-performing athletes are confronted with sport-specific stressors (e.g., injuries, performance setbacks, end of career) that might increase their vulnerability for athletic burnout and psychological impairments^8^. Beyond chronic stress, other factors seem to explain the development of burnout symptoms in athletes, which have been incorporated in the integrated model of athlete burnout by Gustafsson et al.^9^. This model integrates previous empirical findings as well as assumptions of earlier theoretical models, like Smith’s cognitive-affective stress model^7^ and the commitment model^6^ by considering three areas that influence early signs of burnout and burnout itself: (1) major antecedents, (2) entrapment, and (3) personality, coping, and environment. Additionally the model links burnout to further maladaptive consequences like withdrawal^9^.

More precisely, antecedents include, for instance, perceived stress and the experience of general and sport-related stressors that have previously been linked to athletic burnout^10^, as well as excessive effort as a part of overcommitment. Overcommitment is defined as the tendency to overextend oneself regardless of one’s own resources^11^. This personality trait has two opposing sides, as it contributes to success and high performance in competitive sports, on the one hand, but, on the other hand, has been related to burnout symptoms in athletes^1^. The decisive factor in this case is whether commitment is based on enjoyment or entrapment, with entrapment being associated with athletic burnout^12^. Entrapment includes, for example, unidimensional athletic identity and high investments, which are supposed to explain why athletes thrust themselves into burnout and restrain them from leaving the sport^9^. Regarding athletic identity, however, empirical findings are mixed suggesting either a negative or a positive relationship with burnout symptoms^9^. Finally, the area of personality, coping, and environment comprises several factors that make athletes more vulnerable to develop burnout symptoms: perfectionism, trait anxiety, low social support, and lack of coping skills^2,9^. This area is also associated with protective factors that help to prevent athletes from developing burnout symptoms. Interestingly, these protective factors partly overlap with resilience promoting factors identified in the general population^13^: higher trait resilience^14^, self-esteem^15^, self-efficacy expectancy^16^, self-compassion^17^, sense of coherence^18^, and social support^12^. In athletes, the latter is potentially conveyed by a functional and positive coach-athlete relationship^12^.

Although not mentioned explicitly in Gustafsson’s model^9^, empirical research has identified specific cognitive styles in relation to burnout vulnerability and previous work also suggests an important link between cognitive styles and coping styles in predicting, e.g., symptoms of depression and anxiety^19^. With respect to athletes, Turner and Moore^20^ showed that irrational beliefs are linked to emotional and physical exhaustion. For rumination, McMillen^21^ found positive associations with all dimensions of athletic burnout among collegiate athletes. Therefore, investigation of sport-specific rumination as a vulnerability factor for the development of burnout symptoms, particularly in competitive athletes in comparison to leisure athletes, seems worthwhile. In that regard, performance worries, situational irrelevant thoughts and thoughts of escape from competition have been linked to concentration difficulties which in turn can be seen as early symptoms of burnout^9,22^ Outside of the athletic context, studies from the workplace have shown that other maladaptive cognitive styles are related to burnout. For instance, social comparison is a ubiquitous process in competitive sport and in the non-athletic context, a social comparison orientation (i.e., the evaluation of one’s own characteristics in comparison to others) has been associated with higher levels of burnout^23^. Similarly, the fear of negative evaluation has been shown to mediate the relationship between stress and psychopathological symptoms, like depression and anxiety in an academic context^24^. Both cognitive styles appear to be particularly relevant for the athletic context given that benchmarking against other athletes and the evaluation of athletic performance are inherent to competitive sport. However, neither a social comparison orientation nor the fear of negative evaluation have been investigated in the context of athletic burnout, so far.

In addition to the influencing factors of athletic burnout, it is also particularly important to consider its consequences and their predictors. Gustafsson et al.^9^ postulated several sports- and physical health-related consequences of athletic burnout (e.g., withdrawal, long term performance impairment, immune dysfunction), athletic burnout also has negative effects on mental health outcomes, like depression or anxiety, as well as on life satisfaction^3^. Importantly, DeFreese and Smith^25^ reported that the effect of athletic burnout on life satisfaction is no uniform and linear relationship, but that it greatly differs interpersonally. Sport-specific characteristics (e.g., performance level, degree of a sport-related life orientation) and general coping mechanisms have been proposed as conceivable moderators for the relationship between athletic burnout and mental health-associated outcomes (e.g., life satisfaction). However, empirical evidence in athletes is missing to date. In medical students, Wang et al.^4^ observed a significant mediating effect of resilience, as assessed by the Connor-Davidson-Resilience Scale^26^ (CD-RISC), on the relationship between academic burnout and life satisfaction. Beyond measuring stress coping ability in general, as covered by the CD-RISC, the assessment of different coping resources separately might provide a more sophisticated picture that would also allow deriving more specific intervention approaches to alleviate burnout risk. In the general population, the positive influence of several temporally stable and cross-situational stress coping resources on mental health has been shown^27^: Sense of coherence as an adaptive orientation within one’s personality that incorporates comprehensibility, manageability, and meaningfulness of an adverse^28,29^, self-compassion as caring and kind attitude towards oneself when dealing with stressful situations^30^, general-self-efficacy as a stable and wide sense of personal competence to effectively handle a range of stressful and challenging situations^31^, and the ability to bounce back or recover from stress by returning to the previous functioning level^32^.

Based on the above-described theoretical models and empirical evidence as well as the identified research gaps, the present study pursued two main research objectives: First, we investigated general and sport-specific predictors of athletic burnout. We included predictors from the three areas of Gustafsson’s integrated model of athlete burnout^9^, that is, major antecedents (here: stress), entrapment (here, e.g., athletic identity), and personality, coping, and environment (here, e.g., resilience and coach-athlete relationship), and coping-related predictors that have been discussed lately (i.e., cognitive styles). Second, we examined possible moderators, namely, the athlete’s performance level and the above-described stress coping resources (ability to bounce back, sense of coherence, self-efficacy, and self-compassion), that could moderate the relationship between athletic burnout and life satisfaction. Given the differences in the prioritization of sports over other areas of life due to different performance levels and an identity foreclosure with a lack of life experiences outside the sporting context^33^, we directly compared athletes at different performance levels (competitive vs. leisure sports) for both research questions.

## Methods

### Participants and procedure

The dataset used for the present analyses was taken from a larger study that focused on athletes’ cognitions during competitions and that followed a cross-sectional design with a single assessment point. Data collection was conducted via SoSci-Survey^34^ and included an online questionnaire battery with 23 questionnaires (249 items altogether). Completion of the whole questionnaire battery lasted about 35 min. The study was conducted in compliance with the Declaration of Helsinki^35^. The Review Board of the Institute of Psychology at the Johannes Gutenberg-University Mainz has approved the study protocol.

The recruitment procedure of the present study followed a convenient sampling approach. In that regard, athletes from various team and individual sports throughout Germany were contacted through e-mail via their respective clubs or sports associations as well as through social media channels and private contacts. In addition, sports students from different universities were invited via e-mail through their respective academic offices. Inclusion criteria were a minimum age of 16, fluent German skills and active as well as regular participation in sports competitions. Participants were informed about the nature and the procedure of the study and gave written informed consent before completing the questionnaires. Participation was voluntary and athletes received 15€ as compensation, which they could donate in full or in part to the Robert Enke Foundation.

Data from 434 participants were collected. For the present analyses, we did not perform an a priori power and respective sample size calculation, as the dataset was taken from a larger study as outlined above where an a priori power calculation had already been conducted. Data of participants who did not answer carefully (automatically generated variables from SoSci-Survey that identify a too fast processing time: TIME_RSI ≥ 2 and DEG_TIME > 100 considered as noticeable; n = 8;^34^), had missing values (n = 1) or claimed not to take part in competitions (n = 3) were excluded from analyses, resulting in a final sample size of *N* = 422 (M_age_ = 23.65, range = 16–67; 43% female, 57% male). To analyze the differences between competitive and leisure athletes, participants were divided into these two groups according to their statement to the question regarding their performance level. Athletes who reported to be professional, high-performance, or competitive athletes were assigned to the competitive sports group and those who reported to be amateur athletes were allocated to the leisure sports group.

Table 1 provides an overview of the demographic data and characteristics of the sample, divided into competitive sports and leisure athletes.

**Table 1:** Demographic data and characteristics of the sample.

	Competitive sports					Leisure sports				
	N	%	M	SD	Range	N	%	M	SD	Range
Age	–	–	22.59	6.38	16–66	–	–	26.88	10.53	17–67
*Sex*	318	100	–	–		104	100	–	–	
Female	130	40.88		–	–		51	49.04	–	
Male	188	59.12		–	–		53	50.96	–	
Training sessions per week	–	–	6.92	2.99		–	–	4.68	2.03	
Hours of sport per week	316*	–	12.02	5.89		101*	–	8.35	5.63	
Number of years conducting the respective sport	–	–	12.17	5.99		–	–	14.35	7.15	
Individual sports**	176	55.35				33	31.73			
Team sports***	142	44.65				71	68.27			
*Participation in competition*										
Regularly	267	83.96				67	64.42			
Now and then	47	14.78				27	25.96			
Very rarely	4	1.26				10	9.62			
Performance level										
Professional level	5	1.57								
High-performance level	55	17.30								
Competitive level	258	81.13								
Amateur level						104	100			
*Squad level*										
Highest national level or Olympic squad	32	10.06								
Second highest national level or perspective or supplementary squad	41	12.89				2	1.92			
Third highest national level or junior squad	39	12.26				2	1.92			
Fourth highest national level or national squad	91	28.62				4	3.85			
Other lower competition level or other squad status	107	33.65				85	81.73			
No competition level or squad status	8	2.52				11	10.58			

N = number, % = percentage, M = mean, SD = standard deviation, *unrealistic data (> 48 h/week) were excluded **Competitive sports including athletics n = 35; swimming n = 51; Triathlon n = 28; gymnastics n =11; running n = 10; canoeing n = 7; tennis n = 6; combat sports n = 4; powerlifting n = 4; riding n = 3; speedskating n = 3; weightlifting n = 3; crossfit n = 2; rowing n = 2; table tennis n = 1; wheel gymnastics n = 1; sport shooting n = 1; Parachute accuracy landing n = 1; biathlon n = 1; waterskiing n =1; bike sports n = 1. Leisure sports including athletics n = 2; swimming n = 2; triathlon n = 6; gymnastics n = 1; running n = 5; canoeing n = 2; tennis n = 3; combat sports n = 2; riding n = 1; weightlifting n = 1; rowing n = 1; table tennis n = 1; wheel gymnastics n = 1; sport shooting n = 1; bike sports n = 1; billiard n = 2; rhythmic gymnastics n = 1.

^***^Competitive sports including handball n = 41; soccer n = 27; hockey n = 26; volleyball n = 14; basketball n = 10; cheerleading n = 8; Lacross n = 3; vault n = 3; badminton n = 2; pétanque n = 2; canoe polo n = 2; rowing n = 2; fistball n = 1. Leisure sports including handball n = 23; soccer n = 29; hockey n = 1; volleyball n = 6; basketball n = 2; Lacross n = 1; vault n = 1; badminton n = 2; pétanque n = 1; futsal n = 1; belly dancing n = 1; billiard n =1.

### Measures

In the following, the self-report measures used in this study are described in more detail. All questionnaires, questions, and statements were presented in German (for a reference list of the original versions of the questionnaires used please refer to the Supplemental Material). In all questionnaires the scale mean was used for further calculations. Mean, standard deviations, and range of all measures for competitive and leisure athletes as well as the scales’ or subscales’ Cronbach’s alpha (α) of the current sample are shown in Table 2. Additionally, whenever the scales’ or subscales’ Cronbach’s alpha (α) of the German version questionnaire are available, they are reported in Table 2 and are at least acceptable (α > 0.6). Further, whenever we have used the translated versions, reliability and validity of the original questionnaires are reported in the Supplemental Material (Table S2).

**Table 2:** Mean (M), Standard deviation (SD), 95% confidence interval, and range of the used measures and scales’ and subscales’s Cronbach’s alpha (α) of the German version questionnaire (GVQ) and the current sample (CS).

	Questionnaire	α_GVQ_	α_CS_*	Competitive sports			Leisure sports		
				M (SD)	95% CI	range	M (SD)	95% CI	range
Athletic burnout	ABQ								
Athletic burnout: Physical/ emotional exhaustion**		0.80	0.88	2.65 (0.79)	[2.57, 2.74]	1–5	2.24 (0.75)	[2.10, 2.39]	1–4.8
Athletic burnout: Sport devaluation		0.78	0.77	2.30 (0.86)	[2.20, 2.40]	1–5	2.51 (0.82)	[2.35, 2.67]	1–4.4
Athletic burnout: Reduced sense of accomplishment		0.78	0.79	2.61 (0.76)	[2.53, 2.69]	1–4.6	2.72 (0.66)	[2.59, 2.85]	1.6–4.2
*General predictors*									
Ability to bounce back	BRS	0.85	0.82	3.31 (0.77)	[3.23, 3.40]	1.17–5	3.26 (0.71)	[3.12, 3.40]	1–4.67
Fear of negative evaluation	FNE-K	0.94	0.95	3.17 (0.99)	[3.06, 3.28]	1–5	3.19 (0.92)	[3.01, 3.36]	1.08–5
Satisfaction with life				7.87 (2.13)	[7.64, 8.11]	1–11	7.51 (2.02)	[7.12, 7.90]	3–11
Self-compassion	SCS-D short	0.91	0.85	3.04 (0.62)	[2.98, 3.11]	1.42–4.92	3.02 (0.68)	[2.89, 3.16]	1.33–4.5
Self-efficacy	SWE	0.92	0.84	2.90 (0.41)	[2.86, 2.95]	1–4	2.82 (0.38)	[2.75, 2.90]	1.6–3.7
Self-esteem				3.53 (0.99)	[3.42, 3.64]	1–5	3.31 (0.93)	[3.13, 3.49]	1–5
Sense of coherence	SOC-L9	0.87	0.86	4.84 (1.04)	[4.73, 4.96]	1.33–6.78	4.72 (1.00)	[4.53, 4.92]	2–6.56
Stress	PSQ-20	0.92	0.92	2.28 (0.49)	[2.22, 2.33]	1–3.65	2.27 (0.52)	[2.17, 2.37]	1.4–3.8
Social comparison orientation	SCS								
Social comparison orientation: Ability		NA	0.77	3.50 (0.93)	[3.40, 3.60]	1–5	3.47 (0.93)	[3.29, 3.66]	1–5
Social comparison orientation: Opinion		NA	0.73	3.43 (0.91)	[3.26, 3.47]	1–5	3.43 (0.91)	[3.17, 3.51]	1–5
*Sport-related predictors*									
Athletic identity**	AIMS-D	0.74	0.78	5.13 (0.84)	[5.03, 5.22]	1.86–7	4.62 (0.92)	[4.44, 4.80]	2.43–6.86
Coach-athlete relationship	CART-Q								
Coach-athlete relationship: Commitment		0.80	0.82	5.31 (1.33)	[5.16, 5.45]	1–7	4.95 (1.36)	[4.68, 5.21]	1.67–7
Coach-athlete relationship: Closeness		0.86	0.87	5.94 (1.11)	[5.92, 6.06]	1–7	5.69 (1.07)	[5.48, 5.90]	2.5–7
Coach-athlete relationship: Complementarity**		0.85	0.86	5.94 (1.01)	[5.83, 6.05]	1–7	5.60 (1.08)	[5.39, 5.81]	1.75–7
Cognitive interference	TOQS	0.89	0.89	2.51 (0.93)	[2.41, 2.62]	1–5.58	2.54 (0.79)	[2.38, 2.69]	1.22–4.66
Excessive effort**	EESS	0.24–0.69	0.84	3.22 (0.52)	[3.16, 3.27]	1.94–4.67	3.01 (0.51)	[2.91, 3.11]	1.72–4.39
Interpersonal satisfaction		NA	0.84	5.77 (1.25)	[5.63, 5.90]	1–7	5.43 (1.19)	[5.20, 5.66]	2.5–7
Irrational beliefs	iPBI-2	0.92^57^	0.89	3.44 (0.55)	[3.38, 3.50]	1–4.9	3.38 (0.58)	[3.26, 3.49]	1.35–4.6
Satisfaction with coach and training		NA	0.81	5.47 (1.35)	[5.35, 5.64]	1–7	5.36 (1.23)	[5.12, 5.60]	1.5–7
Sport-specific rumination	SCRS	0.92	0.91	2.58 (0.86)	[2.48, 2.67]	1–5	2.60 (0.83)	[2.44, 2.77]	1–4.63

*Total sample included, **indicates significant differences between groups, NA = not available

#### Outcome measure: athletic burnout

To measure clinically relevant athletic burnout symptoms a German version of the Athlete Burnout Questionnaire^36^ (ABQ) was used. It consists of 15 items with each five statements measuring the construct of physical and emotional exhaustion (e.g., “I feel overly tired from my sport participation”), sport devaluation (e.g., “I don’t care as much about my sport performance as I used to do”) and reduced sense of accomplishment (e.g., “I am not performing up to my ability in sport”). Statements are rated on a 5-point scale ranging from 1 (almost never) to 5 (almost always). Mean values for each dimension were formed for further calculations, which implies that the possible outcome in all three scales ranges from 1 to 5. The external validity can be presumed to be sufficient due to the significant correlation with domain-unspecific burnout symptoms (*r* = 0.31 to 0.52)^36^.

#### General predictors

**Ability to bounce back.** The ability to bounce back was assessed using the German Brief Resilience Scale^37^ (BRS). It consists of six items that describe statements about a person’s ability to bounce back (e.g., “I tend to bounce back quickly after hard times”). Items are rated on a 5-point scale from 1 (strongly disagree) to 5 (strongly agree). Sufficient external validity can be assumed due to the significant correlations with the following constructs: optimism (*r* = 0.51), self-efficacy (*r* = 0.51), and internal locus of control (*r* = 0.45)^37^.

**Fear of negative evaluation.** The German version of the Brief Fear of Negative Evaluation Scale (FNE-K;^38^) was used to assess one’s fear of negative social evaluation by others. The scale includes 12 statements (e.g., “I worry about what other people will think of me even when I know it doesn’t make any differences”) that are scored on a 5-point scale ranging from 1 (not at all characteristic of me) to 5 (extremely characteristic of me).

**Life satisfaction.** Satisfaction with life was assessed with a single-item measure^39^. Participants indicated their agreement to the statement “All in all, how satisfied are you with your life at the moment?” on a 11-point scale (0 = not satisfied at all; 10 = completely satisfied). External validity can be assumed to be sufficient due to the significant correlations with the following constructs: self-worth (*r* = 0.49) and domain-specific life satisfaction (i.e. work (*r* = 0.48), health (*r* = 0.48), partnership (*r* = 0.32))^39^.

**Sense of coherence.** The Sense of Coherence Leipzig Short Scale^40^ (SOC-L9) was used to measure a person’s sense of coherence. The SOC-L9 has nine items (e.g., “How often are your feelings and ideas all mixed up?”). Items are rated on a 7-point scale with two anchoring verbal responses. Sufficient external validity can be presumed due to the significant negative correlations with subjective body complaints (*r* = −0.36) and somatoform symptoms (*r* = −0.33)^40^.

**Self-compassion.** The German version of the short version of the Self-Compassion Scale^41^ (SCS-D-short version) was used to assess a participant’s positive attitude towards itself when things go badly. The scale consists of 12 statements (e.g., “I try to see my failings as part of the human condition”) that are scored on a 5-point scale ranging from 1 (almost never) to 5 (almost always). The external validity can be considered sufficient due to the significant correlations with the constructs self-worth (*r* = 0.75) and meta mood trait (*r* = 0.29–0.72)^41^.

**Self-efficacy.** General self-efficacy as an optimistic perceived competence to overcome difficult situation and still attributing the success to the own competence was assessed with the scale of general perceived self-efficacy^42^ (GSE). The scale consists of 10 items (e.g., “I take a relaxed approach to difficulties because I can always trust my abilities”) and is rated on a 4-point scale (1 = not at all true; 4= completely true). Sufficient external validity can be assumed due to the significant correlation (*r* = 0.68) with resilience^42^.

**Self-esteem.** A person’s self-esteem was measured by the German Single-Item Self-Esteem Scale^43^ (G-SISE). Participants indicated their agreement to the statement “I have a high self-esteem” on a 5-point scale ranging from 1 (not very true of me) to 5 (very true of me). The external validity can be assumed to be sufficient due to the significant correlation to another self-esteem measure (*r* = 0.75)^43^.

**Social comparison orientations.** An athletes’ social comparison orientation was assessed by the shortened German version of the Iowa-Netherlands Comparison Orientation Measure^44^ (INCOM). It consists of six items, measuring two core dimensions: ability (3 items, e.g., “I always pay a lot of attention to how I do things compared with how others do things”), and opinion (3 items, e.g., “I often try to find out what others think who face similar problems as I face”). Items were rated on a 5-point scale (1= I disagree strongly, 5= I agree strongly). Sufficient external validity can be assumed due to the non-significant correlations with the following constructs: life satisfaction (*r* = −0.09) and yesterday’s affection (*r* = 0.13)^44^.

**Stress.** The subjective experienced stress of the participants was measured by the Perceived Stress Questionnaire^45^ (PSQ-20). The questionnaire includes four subscales (demands, joy, tension, and worries) and a global mean that was used in this study. Participants indicated their agreement to the statements (e.g., “You feel that too many demands are being made on you”) on a 4-point scale ranging from 1 (almost never) to 4 (most of the time). The external validity can be assumed to be sufficient due to the significant correlation to another chronic stress measure (*r* = 0.52 to 0.81)^45^.

#### Sport-related predictors

**Athletic identity.** The German version of the Athletic Identity Measurement Scale^46^ (AIMS-D) consists of 10 items that describe social, cognitive, and affective aspects of athletic identity (e.g., “I consider myself an athlete”). Items are rated on a 7-point scale of 1 (strongly disagree) to 7 (strongly agree). Sufficient external validity can be assumed due to the significant correlations with the following constructs: training effort (*r* = 0.23) and satisfaction with life in sport (*r* = 0.22)^46^.

**Coach-athlete relationship**. The athlete version of the Coach-Athlete Relationship Questionnaire^47^ (CART-Q) was used to measure the nature of the coach-athlete relationship on three dimensions: commitment, closeness, and complementarity. The CART-Q consists of 11 items, three statements measuring the construct of commitment (e.g., “I feel committed to my coach”), and each 4 statements the construct of closeness (e.g., “I like my coach”), and complementarity (e.g., “When I am coached by my coach, I am ready to do my best”). Items are rated on a 7-point scale ranging from 1 (strongly disagree) to 7 (strongly agree). The external validity can be assumed to be sufficient due to significant correlations with the following constructs: athlete satisfaction (*r* = 0.69–0.79) and general relationship satisfaction (*r* = 0.65–0.72)^47^.

**Cognitive interference.** With the Thought Occurrence Questionnaire for Sport^48^ (TOQS) athletes’ cognitive interference during competitions was measured. It consists of 17 items starting with “during the competition I had thoughts…” and measuring cognitive interference on three dimensions: Task-Related Worries (6 items, e.g., “… about previous mistakes I have made”), Task-Irrelevant Thoughts (5 items, e.g., “…about what I’m going to do later in the day”), and Thoughts of Escape (6 items, e.g., “… that I want to quit”). Items were rated on a 7-point scale ranging from 1 (almost never) to 7 (almost always). In this paper, the subscale averages are summed up. The external validity can be assumed to be sufficient due to significant correlations with the following constructs: concentration problems (*r* = 0.49) and cognitive competition anxiety (*r* = 0.53)^48^.

**Excessive effort.** The German Excessive Effort in Sport Scale^11^ (EESS) was used to assess an athlete’s excessive effort based on various behaviors and experiences in sports. The scale consists of 18 items (e.g., “I wish to be recognized for my commitment in sports”) that are rated on a 5-point scale of 1 (not at all true) to 5 (completely true).

**Interpersonal satisfaction.** Athletes’ interpersonal satisfaction was measured by two questions (according to Jowett and Ntoumanis^49^; e.g. “Do you feel satisfied by your overall coach-athlete relationship?” and “Do you think your athlete/coach feels satisfied by your coach–athlete relationship as a whole?”)^49^, rated on a 7-point scale ranged from 1 (Not-at-all) to 7 (extremely).

**Irrational beliefs**. The sport version of the irrational Performance Beliefs Inventory^50^ (G-iPBI-2) was used to assess athletes’ irrational performance beliefs. It consists of 20 items and measures four core irrational beliefs: demands, awfulizing, low frustration tolerance, and depreciation. All items were rated on a 5-point scale ranging from 1 (strongly disagree) to 5 (strongly agree). In this paper the subscale averages were summed up for a composite score. Sufficient external validity can be assumed due to the significant correlations with dysfunctional attitudes, i.e. perfectionism (*r* = 0.57) and dependency (*r* = 0.61)^50^.

**Satisfaction with coach and training.** Athletes’ satisfaction with the coach and training they received was assessed with two statements according to Alfermann et al.^51^ (e.g. “How satisfied are you with your coach” and “How satisfied are you with the training you receive?”), each rated on a 7-point scale (1 = not at all satisfied, 7 = very satisfied). External validity can be presumed as there are no significant correlations with specific motivational climate (e.g., mastery climate (*r* = 0.08) or performance climate (*r* = −0.02))^51^.

**Sport-specific rumination.** The Sports Competition Rumination Scale^52^ (SCRS) consists of eight items measuring sport-specific ruminative thoughts about competition-related problems (e.g., “I can’t stop thinking about competition-related problems”). Items are scored on a 5-point scale ranging from 1 (not true at all) to 5 (completely true). Sufficient external validity can be assumed due to significant correlations with other rumination measures (*r* = 0.53–0.63)^52^.

### Data analyses

Data analyses were performed with R and RStudio^53,54^. To answer the first research question of the present study, namely general and sport-specific predictors of athletic burnout, exploratory factor analysis (EFA; R-packages *nFactors*, *GPArotation*) to identify latent variables and regression analyses within structural equation models (SEM; R-Packages *lavaan*) were carried out. To address the second research question, i.e. examination of possible moderators (such as athlete’s performance level and the stress coping resources) that could determine the relationship between athletic burnout and life satisfaction, moderation analyses were conducted.

## Research question 1: regression analyses within SEMs

**Determination of latent variables and set-up of measurement model.** Strong correlations between questionnaires result in multicollinearity, which would violate the requirements for regression analyses. To solve multicollinearity problems, several options have been suggested. Among these options, we chose to reduce predictors based on exploratory factor analysis and use linear SEM, subsequently, to confirm the measurement model. Details on the statistical analyses are described in the following.

Since the latent variables used for the regression analyses within SEM were determined exploratory, we created two subsamples with an equal amount of competitive and leisure athletes in both subsamples: Subsample 1 was used to develop a measurement model, whereas subsample 2 was used to evaluate the developed measurement model. Initially, the overall sample was divided into athletes who claimed to do leisure sports (*N* = 104) and those who stated to do competitive sports (*N* = 319). The group of leisure athletes were randomly assigned to subsample 1 and 2. To have an equal sample size for competitive and leisure athletes in both subsamples, 104 competitive athletes were randomly selected and again randomly assigned to subsamples 1 and 2. Thus, each subsample finally consisted of a total of 104 athletes, 52 leisure athletes and 52 competitive athletes.

To find suitable latent variables, an EFA with subsample 1 was performed, including the following measures: *ability to bounce back, fear of negative evaluation, satisfaction with life, self-compassion, self-efficacy, self-esteem, sense of coherence, stress, social comparison orientation ability and opinion, athletic identity, coach-athlete relationship commitment, closeness, and complementarity, cognitive interference, excessive effort, interpersonal satisfaction, irrational beliefs, satisfaction with coach and training,* and *sport-specific rumination*. Due to the potential interdependence of the constructs, a factor analysis with an oblique rotation (oblimin) was carried out^55^. The factors identified in the EFA were used as latent variables for the measurement model. First, a SEM with the developed measurement model was conducted in subsample 1, second the same measurement model was applied to subsample 2, to validate the measurement model in an independent sample. Both SEMs were performed with maximum likelihood estimation with robust (Huber-White) standard errors (MLR). To evaluate how well the proposed model aligns with the observed data, the root mean square error of approximation (RMSEA), the standardized root mean square residuals (SRMR), the *Χ*^2^/df, and Comparative Fit Index (CFI) are reported in addition to the *Χ*^2^-statistic, which is sensitive to sample size and complexity of a model^56^. The *p* value of *Χ*^2^-Test should be > 0.05 to not reject the hypothesis of a perfect fit. *Χ*^2^/df should be < 3.0 for an acceptable fit^57^. The RMSEA should be < 0.08 for an acceptable fit and < 0.05 for a good fit^57^. The SRMR should also be < 0.08 for an acceptable fit to the data^58^. The CFI has a range from 0 to 1, with values > 0.90 and > 0.95 are considered as an acceptable and an excellent fit, respectively^59^. To achieve a good fitting model, at least two fit indices should have an acceptable fit^58^. The fit indices of both models from subsamples 1 and 2 were compared to determine whether the developed latent variables are found in the initial as well as the independent subsample.

**Regression analyses within SEM**. The above-explained identification of latent variables was used to reduce dimensions and thereby potential multicollinearity; however, questionnaire or single-item scores excluded during EFA were nevertheless deemed relevant predictors and were therefore included as manifest variables in the regression analysis within SEM, in addition to the latent variables. They were used as predictor variables, whereas the burnout dimensions (physical/emotional exhaustion, sport devaluation, and reduced sense of accomplishment) served as outcome variables. For each performance level (competitive vs. leisure sports), a SEM was conducted with all predictor variables included at once. Both SEMs were performed with MLR. To evaluate how well the proposed model aligns with the observed data, the same indices were used as for model development (RMSEA, SRMS, *Χ*^2^/df, CFI, *Χ*^2^-statistic; see above). R^2^ were reported for every burnout dimensions separately.

## Research question 2: moderation analysis

First, performance level was tested as a moderator in the relationship between athletic burnout and life satisfaction. A linear regression analysis was performed, using life satisfaction as the outcome variable and all burnout dimensions, performance level, as well as the interactions between the burnout dimensions and the performance level as predictors.

Second, further moderation analyses with the stress coping mechanisms (i.e., sense of coherence, ability to bounce back, self-efficacy, and self-compassion) as moderators were conducted separately according to performance level. One analysis was performed per moderator and significance levels were corrected by the number of moderation analyses performed (significance level *p* < 0.05 were divided by 4, therefore corrected significance level was *p* < 0.0125). For significant moderators, Johnson-Neyman analyses were conducted to examine the range of significance.

## Results

### Descriptive results

Table 2 summarizes the descriptive statistics of the different questionnaires separately for competitive and leisure athletes. Regarding the main outcome variables, both groups indicate medium athletic burnout levels. However, in both groups, the range is fully utilized indicating burnout for some athletes. In addition, there are no group differences regarding the average level of athletic burnout symptoms, except of *physical/ emotional exhaustion*, where competitive athletes reported higher levels than leisure athletes. For the non-sport-specific predictor variables, there are again no group differences. Except of *athletic identity*, *excessive effort,* and *coach-athlete relationship: complementarity*, where competitive athletes again reported higher values than leisure athletes, this applies for the sport-specific predictor variables, too, indicating that the two groups are comparable in these dimensions. Differences in the subsequent analyses, therefore, cannot be attributed to general differences between competitive and leisure athletes.

### Exploratory factor analysis

Due to the high correlations between some predictors (e.g., CART-Q and satisfaction with coach/training; see Table S1 in the Supplemental Material), multicollinearity could pose a problem for regression analyses. Therefore, exploratory factor analysis was employed to reduce dimensions for subsequent analyses and to create latent factors. Exploratory factor analysis with subsample 1 was carried out to identify variables loading on the same psychological construct. KMO of all variables were above 0.50 (KMO overall = 0.82) and Bartlett’s Tests of Sphericity was significant (*χ*^2^(153) = 1004.53, *p* < 0.001), indicating that the data was suitable for factor analysis^60^. Due to the potential interdependence of the constructs, a factor analysis with an oblique rotation (oblimin) and the principal factor solution was computed^55^. According to Tabachnick and Fidell^55^ the following criteria for variable extraction were used: (1) eigenvalues greater than 1.0, (2) per component a minimum of 5% explained variance, and (3) significant unique loadings (sample size = 100, therefore Λ ≥ 0.55), and cross-loading differences ≥ 0.10. Variables that do not reach the proposed criteria were extracted successively and new factor analyses were computed. Due to poor factor loadings (Λ < 0.55), *irrational beliefs, cognitive interference, sport-specific rumination, athletic identity, excessive effort,* and *self-esteem* were excluded successively. The results of the exploratory factor analysis with the variables’ loadings, the eigenvalues, and the percentage of variance explained by each component are shown in Table 3. Scree-plot analysis and parallel analysis finally resulted in a three-factor structure that accounted for a total of 65.1% of the overall variance. The three factors could be best described as “coach athlete relationship”, “resilience” and “social comparison”. Multivariate normal distribution of the variables cannot be assumed due to the significant results of the Mardia Skewness and Kurtosis Test (Mardia Skewness: *β*_*s*_ = 2357.75, *p* < 0.001; Mardia Kurtosis: *β*_*k*_ = 19.38,* p* < 0.001). Thus, in the following, robust measurement methods were used.

**Table 3:** Extracted factors of the exploratory factor analysis with variables’ loadings, eigenvalues, and variance explained by each factor.

	Coach-athlete relationship	Resilience	Social comparison
Coach-athlete relationship: closeness	0.874		
Coach-athlete relationship: commitment	0.886		
Coach-athlete relationship: complementarity	0.835		
Satisfaction with coach and training	0.821		
Interpersonal satisfaction	0.777		
Sense of coherence		0.831	
Self-efficacy		0.690	
Ability to bounce back		0.708	
Self-compassion		0.744	
Social comparison orientations: ability			0.847
Social comparison orientations: opinion			0.735
Fear of negative evaluation			0.652
Eigenvalues	3.582	2.403	1.831
Variance explained (%)	46	31	23

Loadings < 0.30 are not displayed.

### Latent factors

The components revealed by the factor analysis were used to create latent factors for the measurement model. To find meaningful latent factors, the factor loadings, the Average Variance Extracted (AVE) as an indicator for convergent validity and composite reliability were checked. Table 4 displays the estimates, standard deviation, *p* values, and factor loadings of the indicator variables used for the latent factors and the AVE and composite reliability (CR) for each latent factor in subsample 1. All indicators achieved factor loadings > 0.55. Regarding the latent factors, all AVE’s were > 0.5, indicating an acceptable level of convergent validity and all CR’s were > 0.7, indicating acceptable reliability^61,62^.

**Table 4:** The latent factors of subsample 1 with the estimates, standard error (SE), *p* values, and factor loadings of the indicator variables and the Average Variance Extracted (AVE) and composite reliability (CR) for each latent factor.

	Estimate	SE	*p*	Factor loadings	AVE	CR
*Coach-athlete relationship*					0.707	0.927
Coach-athlete relationship: closeness	1.000			0.886		
Coach-athlete relationship: commitment	1.355	0.101	< 0.001	0.883		
Coach-athlete relationship: complementary	1.168	0.116	< 0.001	0.792		
Satisfaction with coach and training	1.012	0.094	< 0.001	0.853		
Interpersonal satisfaction	1.213	0.121	< 0.001	0.784		
*Resilience*					0.569	0.837
Sense of coherence	1.000			0.832		
Ability to bounce back	0.619	0.090	< 0.001	0.717		
Self-compassion	0.512	0.068	< 0.001	0.738		
Self-efficacy	0.342	0.045	< 0.001	0.724		
*Social comparison*					0.603	0.825
Social comparison orientations: ability	1.000			0.911		
Social comparison orientations: opinion	0.837	0.130	< 0.001	0.704		
Fear of negative evaluation	0.804	0.138	< 0.001	0.695		

To see whether the results of subsample 1 can be replicated, the same measurement model was conducted with subsample 2. The fit indices of both subsamples are shown in Table 5. Both subsamples show an acceptable absolute fit (robust RMSEA < 0.08, *p* > 0.05 indicating that a good fit cannot be ruled out, SRMR < 0.08, Χ^2^/*df* < 3.0) and a good comparative fit (robust CFI > 0.950)^58,63–65^.

**Table 5:** Fit indices of the measurement model in subsample 1 and subsample 2, and the structural equation models of competitive and leisure athletes.

	*Χ*^2^-Test (51)	Robust RMSEA [90%-CI]	SRMR	Robust CFI	Χ^2^/df
Subsample 1	77.061; *p* = 0.011	0.070 [0.034;0.100]; *p* = 0.154	0.069	0.962	1.511
Subsample 2	63.236; *p* = 0.117	0.049 [0.000;0.085]; *p* = 0.487	0.054	0.981	1.589
Competitive sports	315.716; *p* < 0.001	0.065 [0.056;0.075]; *p* = 0.011	0.040	0.946	2.239
Leisure sports	203.139; *p* < 0.001	0.067 [0.045;0.086]; *p* = 0.098	0.054	0.937	1.441

### Regression analysis

**Fig. 1:**
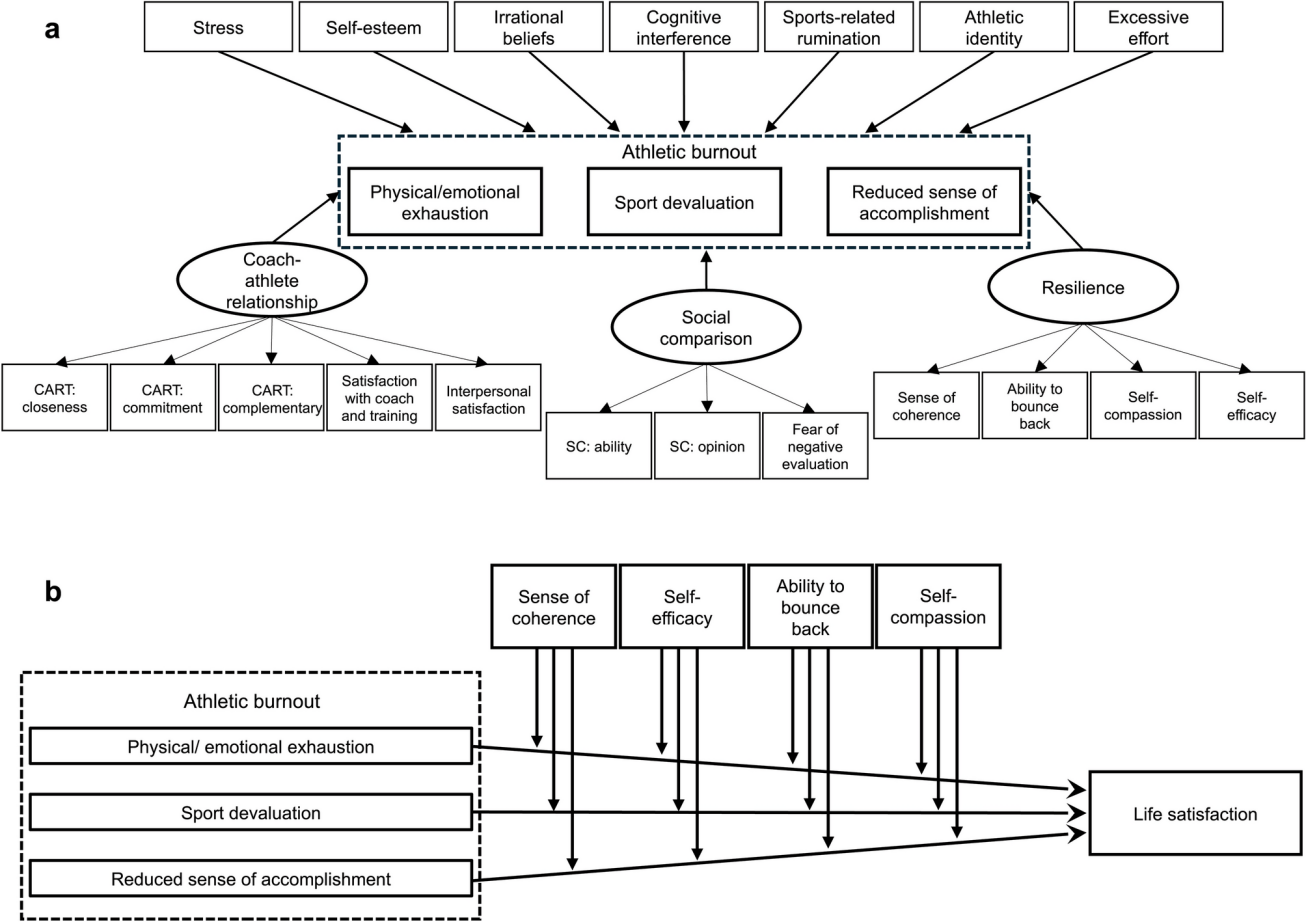
(**a**) Structural equation models used for competitive and leisure athletes (CART = Coach-athlete relationship; SC = Social comparison orientation) and (**b**) moderation analyses with sense of coherence, self-efficacy, ability to bounce back, and self-compassion as moderators.

SEM were developed to test the impact of stressors and stress coping resources on the dimensions of athletes’ burnout. Figure 1a illustrates the model. Two SEMs—one for competitive sports and one for leisure sports—were conducted with the latent variables from the measurement model and the manifest variables (that were excluded during the EFA) and the dimensions of athletes’ burnout as outcome variables. Intercorrelations between the predictors were not permitted. Table 5 shows the fit indices of the complete models. Both SEMs showed an acceptable fit; CFI > 0.9 and RMSEA ≤ 0.08^64,65^.

Table 6 displays the results of the regression analysis within SEM with the estimates, standard errors, and the *p* values for competitive and leisure athletes.

**Table 6:** Regression analyses with structural equation models for competitive and leisure sports with the burnout subdimensions (physical/ emotional exhaustion, sport devaluation, and reduced sense of accomplishment) as outcome variables.

	Competitive sports				Leisure sports			
	Estimate	*SE*	*p*	R^2^	Estimate	*SE*	*p*	R^2^
*Burnout: Physical/ emotional exhaustion*				0.172				0.192
Intercept	2.653	0.044	<0.001**		2.244	0.073	< 0.001**	
Coach-athlete relationship	0.048	0.049	0.322		−0.018	0.076	0.813	
Resilience	0.055	0.127	0.665		−0.266	0.271	0.327	
Social comparison	−0.092	0.089	0.301		−0.085	0.156	0.587	
Stress	0.444	0.151	0.003**		0.215	0.313	0.492	
Self-esteem	0.000	0.058	0.996		0.283	0.092	0.002**	
Irrational beliefs	−0.184	0.107	0.085		0.000	0.143	0.999	
Cognitive interference	0.243	0.054	< 0.001**		0.141	0.091	0.122	
Sport-specific rumination	0.039	0.069	0.568		−0.132	0.120	0.270	
Athletic Identity	0.107	0.055	0.052		0.029	0.077	0.710	
Excessive effort	0.220	0.098	0.026*		0.106	0.174	0.543	
*Burnout: Sport devaluation*				0.284				0.204
Intercept	2.300	0.048	< 0 0.001**		2.513	0.080	< 0.001**	
Coach-athlete relationship	−0.089	0.050	0.075		−0.124	0.080	0.119	
Resilience	0.014	0.135	0.918		−0.002	0.252	0.994	
Social comparison	−0.119	0.089	0.180		0.081	0.181	0.652	
Stress	0.305	0.168	0.070		0.307	0.337	0.363	
Self-esteem	−0.106	0.061	0.081		0.110	0.100	0.271	
Irrational beliefs	−0.111	0.107	0.297		−0.022	0.181	0.905	
Cognitive interference	0.270	0.059	< 0.001**		0.320	0.103	0.002**	
Sport-specific rumination	−0.043	0.064	0.501		0.069	0.098	0.482	
Athletic Identity	−0.302	0.061	< 0.001**		0.013	0.094	0.888	
Excessive effort	0.069	0.097	0.479		0.001	0.172	0.993	
*Burnout: Reduced sense of accomplishment*				0.499				0.598
Intercept	2.609	0.042	< 0.001**		2.721	0.064	< 0.001**	
Coach-athlete relationship	−0.178	0.045	< 0.001**		−0.083	0.058	0.158	
Resilience	−0.245	0.086	0.004**		−0.699	0.237	0.003**	
Social comparison	−0.117	0.070	0.093		−0.041	0.115	0.723	
Stress	0.071	0.106	0.502		−0.459	0.311	0.140	
Self-esteem	−0.071	0.046	0.121		0.098	0.070	0.161	
Irrational beliefs	0.217	0.070	0.002**		0.332	0.112	0.003**	
Cognitive interference	0.192	0.045	< 0.001**		0.160	0.065	0.014*	
Sport-specific rumination	0.105	0.050	0.034*		0.045	0.078	0.563	
Athletic Identity	−0.116	0.038	0.002**		−0.017	0.072	0.810	
Excessive effort	−0.129	0.068	0.055		−0.378	0.100	< 0.001**	

Significant values are in bold.

*p < 0.05. **p < 0.01.

**Competitive athletes**. For the burnout dimension *physical/emotional exhaustion* the regression analysis within SEM revealed the following significant predictors: an athletes’ *cognitive interference* (*p* < 0.001), *stress* (*p* = 0.003) and *excessive effort* (*p* = 0.026). For the burnout dimension *sport devaluation* an athletes’ *cognitive interference* (*p* < 0.001) and *athletic identity* (*p* < 0.001), were identified as significant predictors. And finally, for the burnout dimension *reduced sense of accomplishment*, the predictors *coach-athlete relationship* (*p* < 0.001), *resilience* (*p* = 0.004), *irrational beliefs* (*p* = 0.002), *sport-specific rumination* (*p* = 0.034), and *athletic identity* (*p* = 0.002) reached significance.

**Leisure athletes**. For the burnout dimension *physical/emotional exhaustion* only *self-esteem* (*p* = 0.002) and for the burnout dimension *sport devaluation* only *cognitive interference* (*p* = 0.002) were significant predictors. For the burnout dimension *reduced sense of accomplishment* an athletes’ *resilience* (*p* = 0.003), *irrational beliefs* (*p* = 0.003), *cognitive interference* (*p* = 0.014), and *excessive effort* (*p* < 0.001) were identified as significant predictors.

### Moderation analysis

To check whether the correlations between the subdimensions of burnout and life satisfaction are moderated by the performance level of the athletes (leisure vs. competitive sports), a moderation analysis with the subdimensions of burnout (*physical/emotional exhaustion*, *sport devaluation*, and *reduced sense of accomplishment*) as predictor variables, the *performance level* as the moderator and *life satisfaction* as the outcome variable was conducted. Analysis did not show a significant moderation effect of performance level on the effect between burnout dimensions and *life satisfaction* (*p* > 0.05; *F*(7,414) = 11.29, *p* < 0.001, *R*^*2*^* =* 0.146). According to Hayes^66^, the interaction term was dropped from the model and a new simple effect model was conducted. The simple effect model revealed a significant relationship between *reduced sense of accomplishment* and *life satisfaction* (B= −1.100, *p* < 0.001), indicating a detrimental effect of *reduced sense of accomplishment* on *life satisfaction* for all athletes. *Physical/emotional exhaustion*, *sport devaluation*, and *performance level* did not show significant effects on *life satisfaction* (*p* > 0.05). To see whether the resilience factors moderate this effect or explain the absence of an overall effect of *physical/emotional exhaustion* and *sport devaluation* on *life satisfaction* and to explore whether there are differences in the moderation depending on the *performance level*, further analysis in competitive vs. leisure athletes were conducted separately in both groups.

Figure 1b illustrates the moderation analyses for competitive and leisure athletes. In total, we conducted eight moderation analyses, four for competitive and four for leisure athletes. Each analysis included one moderator (*sense of coherence*, *ability to bounce back*, *self-compassion*, or *self-efficacy*), the burnout dimensions (*physical/emotional exhaustion*, *sport devaluation*, and *reduced sense of accomplishment*) as predictor variables, and *life satisfaction* as the outcome variable. *P* values have been corrected for all analyses within each performance level (*p*_corrected_ < 0.05/4 = 0.0125). Table 7 contains the results of the moderation analysis. For competitive athletes the significant relationship between *reduced sense of accomplishment* and *satisfaction of life* was significantly moderated by *self-compassion* (*p* < 0.001), and *self-efficacy* (*p* = 0.009), whereas no significant moderating effects were found for leisure athletes. For this group, only a significant main effect for *sense of coherence* (*p* < 0.001) was observed indicating higher *satisfaction of life* with a stronger *sense of coherence*.

**Table 7:** Moderation analysis for competitive and leisure athletes, separated by each moderator: sense of coherence, ability to bounce back, self-compassion, and self-efficacy.

	Competitive sports			Leisure sports		
	Estimate	*SE*	*p*_*corrected*_* < 0.0125*	Estimate	*SE*	*p*_*corrected*_* < 0.0125*
*Sense of coherence*						
Intercept	4.578	2.092	0.029	−5.815	3.921	0.141
Physical/ emotional exhaustion (PEX)	−0.276	0.542	0.612	1.835	0.811	0.026
Sport devaluation (SpD)	0.080	0.504	0.874	−0.728	1.036	0.484
Reduced sense of accomplishment (RSoA)	−0.880	0.607	0.148	1.429	1.135	0.211
Sense of Coherence (SoC)	0.790	0.400	0.049	2.740	0.805	< 0.001**
Interaction PEX * SoC	0.040	0.110	0.720	−0.315	0.172	0.070
Interaction SpD * SoC	0.004	0.107	0.972	0.149	0.212	0.484
Interaction RsoA * SoC	0.145	0.121	0.234	−0.338	0.249	0.179
*Ability to bounce back*						
Intercept	9.352	2.004	< 0.001**	3.015	4.691	0.522
Physical/ emotional exhaustion (PEX)	0.049	0.615	0.936	0.390	1.171	0.739
Sport devaluation (SpD)	−0.150	0.600	0.803	1.316	1.477	0.375
Reduced sense of accomplishment (RsoA)	−1.328	0.665	0.047	−0.744	1.573	0.637
Ability to bounce back (AtBB)	0.336	0.571	0.556	1.904	1.346	0.160
Interaction PEX * AtBB	−0.082	0.184	0.655	−0.044	0.353	0.901
Interaction SpD * AtBB	0.053	0.182	0.774	−0.354	0.442	0.425
Interaction RsoA * AtBB	0.166	0.200	0.409	−0.083	0.468	0.859
*Self-compassion*						
Intercept	11.497	2.118	< 0.001**	0.292	4.315	0.946
Physical/ emotional exhaustion (PEX)	−0.242	0.635	0.703	1.233	0.873	0.161
Sport devaluation (SpD)	0.901	0.625	0.150	0.569	1.202	0.637
Reduced sense of accomplishment (RsoA)	−3.512	0.701	< 0.001**	−0.535	1.394	0.702
Self-compassion (SC)	−0.525	0.670	0.434	2.524	1.355	0.066
Interaction PEX * SC	−0.009	0.203	0.966	−0.290	0.304	0.343
Interaction SpD * SC	−0.340	0.207	0.102	−0.220	0.412	0.595
Interaction RsoA * SC	1.041	0.236	< 0.001**	0.053	0.486	0.913
*Self-efficacy*						
Intercept	11.238	3.359	0.001	−6.454	7.341	0.382
Physical/ emotional exhaustion (PEX)	0.201	0.972	0.836	−0.621	2.056	0.763
Sport devaluation (SpD)	0.866	0.975	0.375	0.720	1.897	0.705
Reduced sense of accomplishment (RsoA)	−3.467	1.046	0.001**	3.282	2.002	0.104
Self-efficacy (SEf)	−0.215	1.105	0.836	5.796	2.560	0.026
Interaction PEX * SEf	−0.161	0.332	0.628	0.318	0.729	0.664
Interaction SpD * SEf	−0.310	0.337	0.358	−0.235	0.667	0.725
Interaction RsoA * SEf	0.949	0.363	0.009*	−1.598	0.734	0.032

Significant values are in bold.

*p_corrected_ < 0.0125. **p_corrected_ < 0.0025. Competitive sports: SoC: *F*(7,310) = 36.67, *p* < 0.001, *R*^2^ = 0.441; AtBB:* F*(7,310) = 11.67, *p* < 0.001, *R*^2^ = 0.191; SC: *F*(7,310) = 21.47, *p* < 0.001, *R*^2^ = 0.311; SEf: *F*(7,310) = 12.10, *p* < 0.001, *R*^2^ = 0.197; Leisure sports: SoC: *F*(7,96) = 17.09, *p* < 0.001, *R*^2^ = 0.5223; AtBB:* F*(7,96) = 3.76, *p* = 0.001, *R*^2^ = 0.158; SC: *F*(7,96) = 7.62, *p* < 0.001, *R*^2^ = 0.310; SEf: *F*(7,96) = 4.52, *p* < 0.001, *R*^2^ = 0.193.

The following Johnson-Neyman analyses revealed that for *self-compassion* the interaction is significant outside the interval of 3.06 and 3.93 (range of observed values = [1.42, 4.92]), and for *self-efficacy* the interaction is significant for values < 3.20 (range of observed values = [1.00, 4.00]). For *self-efficacy*, increasing values can buffer the detrimental effect of *reduced sense of accomplishment* on *life satisfaction*. In *self-compassion*, the same pattern can be seen with even a reversed trend with high values of *self-compassion* leading to a beneficial effect of *reduced sense of accomplishment* on *life satisfaction* (For interaction plots please refer to Fig. S1 in the Supplemental Material).

## Discussion

The present study followed two main research questions: First, we identified predictors of athletic burnout according to the integrated model of athlete burnout by Gustafsson et al.^9^ and determined whether predictors differ between competitive and leisure athletes. Second, we examined whether athletes’ performance level and/or stress coping resources are significant moderators of the relationship between dimensions of athletic burnout and life satisfaction. The results of the present study revealed distinct patterns of predictors for the different subdimensions of athletic burnout on the one hand and for the two different performance levels (i.e., competitive vs. leisure sports) on the other. In general, we observed more complex patterns of predictors in competitive as compared to leisure athletes for all three dimensions of athletic burnout; in particular, sport-specific predictors were more relevant in predicting the different dimensions of athletic burnout in competitive athletes than in leisure athletes. Interestingly, in competitive athletes two sport-specific constructs, namely cognitive interference, and athletic identity, significantly predicted all three dimensions of athletic burnout (with one exception, which showed a trend towards significance, see below). Whereas a significant relationship between reduced sense of accomplishment and life satisfaction was observed in both competitive and leisure athletes, only in competitive athletes we found coping resources to significantly moderate the relationship between athletic burnout and life satisfaction.

In the following, the results of the first research question are discussed in more detail. For this purpose, the areas of the integrated model of athletic burnout^9^ ((1) major antecedents, (2) entrapment, and (3) personality, coping, and environment) as well as additional predictors are considered separately with regard to the predictability of the subdimensions of athletic burnout for competitive and leisure athletes.

Regarding major antecedents, stress associated variables (i.e., perceived stress and excessive effort) were positive significant predictors of physical/ emotional exhaustion among competitive athletes and negative predictors of reduced sense of accomplishment among leisure athletes. Our results are partially in line with previous studies showing that stress and stress inducing variables predict higher athletic burnout^10^. However, in the present study this effect was only detectable among competitive athletes. For excessive effort, its significant role in predicting exhaustion in competitive athletes only seems plausible, given the differences in the number of hours trained and competitions completed. Further, although perceived stress was surveyed in general and not sport-related, competitive athletes might have associated the questions more strongly with their sporting activity than leisure athletes, because sport is a more important part of their everyday lives. More specifically, leisure athletes may have reported high levels of perceived stress because they have, for example, a lot of stress at work or in training and this does not affect the sports context in the sense of athletic burnout. Furthermore, for some leisure athletes, sport is probably more of a compensation, whereas for competitive athletes it can be a source of stress. Regarding the negative relationship between excessive effort and reduced sense of accomplishment in leisure athletes, it is important to note the cross-sectional design of the study and the associated impossibility to distinguish between cause and effect. Therefore, it is also possible that a decline in excessive effort is a result of a reduced sense of accomplishment as previous findings suggested with athletic identity^67^.

Concerning the area of entrapment, athletic identity was negatively associated with the burnout dimensions sport devaluation and reduced sense of accomplishment and positively with the burnout dimension physical/ emotional exhaustion, even though it just failed to reach significance for this specific dimension (*p* = 0.052). This pattern was only found in competitive but not leisure athletes. Our finding is in line with previous findings that observed a relationship between athletic identity (especially an unidimensional one) and entrapment in sport, contributing to greater exhaustion in athletes^9^. Also, reduced athletic identity might also result from burnout symptoms in the way that a devaluation of the own sports and a reduced sense of accomplishment and therefore a negative self-evaluation as an athlete leads to a decline in athletic identity^67^. In contrast to competitive athletes, who more likely tend to have a unidimensional sport-related identity, leisure athletes probably exhibit a more multidimensional identity reducing the impact of their athletic identity on burnout symptoms.

Regarding the area of personality, coping, and environment, coach-athlete relationship appeared to be a protective factor for the burnout dimension reduced sense of accomplishment among competitive athletes, which is in line with previous findings about social support^12^. An interplay between different predictors might play a role for the observed differences between competitive and leisure athletes: Due to a higher priority of sports success and, consequently, time spent with training for competitive athletes, the coach-athlete relationship also takes on a more important role in their lives. Thus, a positive interaction with the coach can lead to stronger effects on the well-being of the athletes. In both competitive and leisure athletes, our results additionally show that the latent factor resilience, consisting of the ability to bounce back, self-efficacy, self-compassion, and sense of coherence, is a protective predictor of the subdimension reduced sense of accomplishment, which is partly in line with previous research showing this effect also for other subdimensions (i.e. resilience and sport devaluation^14^) or reporting significant correlations with negative health outcomes^16–18^. Finally, only among leisure athletes, we observed self-esteem to be a positive predictor of the burnout dimension physical/emotional exhaustion, which is in contrast to previous research reporting a negative correlation between athletic burnout and self-esteem^15^. Due to the cross-sectional study design interpretations are speculative and must be followed up with longitudinal studies in the future; however, greater self-esteem might lead to more confidence in one’s own performance which might result in excessive demands. Considering that higher self-esteem is also an aspect of a narcistic personality, it is noteworthy that previous research observed more physical/emotional exhaustion symptoms in narcistic individuals^68^.

In addition to Gustafsson’s model^9^, further predictors of athletic burnout symptoms were investigated in this study. As for irrational beliefs, our results are partly in line with Turner and Moore^20^ who only observed a significant association with the burnout dimension physical/ emotional exhaustion in an elite athlete sample but not with reduced sense of accomplishment as in our study. In our study a trend between irrational beliefs and exhaustion (*p* = 0.085) was detectable among competitive athletes. Since we did not analyze elite athletes and other competitive athletes separately, the lack of significance might be attributed to this mixed sample and should therefore interpreted with caution. Sport-specific rumination was a further cognitive predictor for a reduced sense of accomplishment in competitive athletes. This result is in line with previous findings that suggested a positive link between rumination and burnout^21^. The group of leisure athletes in our study was potentially too heterogeneous regarding the relevance for competitions with respect to further athletic careers and personal athletic goals to reveal a significant effect for that variable on burnout symptoms. Finally, the latent factor social comparison, consisting of social comparison orientation and fear of negative evaluation, failed to be a predictor for any burnout subdimension in both groups of athletes. Although this needs to be investigated further, this dimension could—to put it cautiously—be rather a predictor of other psychological impairments such as anxiety or depression^23,24^ than athletic burnout.

Besides the identification of differentiating predictors of athletic burnout in competitive and leisure athletes, the present study aimed to determine sport-specific and psychological moderators of the relationship between the subdimensions of athletic burnout and life satisfaction. Results showed that only the subdimension reduced sense of accomplishment significantly contributed to a decline in life satisfaction among all athletes, aligning with previous research that reported a significant relationship between athletic burnout and life satisfaction^3^. Moreover, only in competitive athletes, self-efficacy and self-compassion could be found as significant moderators, whereas the ability to bounce back and sense of coherence were non-significant. These findings seem counterintuitive, as the joint analysis showed a negative simple effect of reduced sense of accomplishment on life satisfaction for all athletes; yet, in the separate moderation analyses, self-compassion and self-efficacy mitigated the detrimental effect of a reduced sense of accomplishment on life satisfaction only for competitive athletes. Therefore, it seems reasonable to consider further aspects that seem inherent in competitive sport. For example, competitive athletes may have more experience in dealing with failure in an athletic context compared to leisure athletes, or the greater focus and pressure in competitive sports necessitate more effective coping strategies. Additionally, other individual and contextual factors might be relevant in competitive athletes, which should be considered in future research. Our results therefore open a promising direction for future studies, particularly to investigate aspects of athletes’ self-concept as protective moderators between athletic burnout and life satisfaction as well as group differences in those coping resources between competitive and leisure athletes.

The results of the present study must be interpreted in the light of some limitations. First, as our study is cross-sectional, it is not designed to draw causal conclusions and as pointed out above for some predictors causes and consequences cannot be distinguished. Thus, trajectories and the development of the subdimensions of athletic burnout as well as changes in predictors due to changes in athletic burnout cannot be analyzed with these data. To tackle these questions, cross-lagged panel studies are needed. Second, the division into the two performance groups is quite simplistic rough and based on the athletes’ self-reports and the small number of athletes claimed to do sport on a professional or high-performance level did not allow to further subdivide competitive athletes into further and more fine-grained performance levels. Even though we predefined performance levels within the questionnaire, it seems conceivable that individual expectations of the performance levels influenced the athletes’ statement. Third, future studies should attempt to collect larger samples with equal proportions of athletes of different performance levels to obtain more meaningful results. Fourth, the age range was very broad (16–67 years), which could have potentially led to differences in the predictors of athletic burnout depending on variations in developmental stages and life phases. Future studies should examine different age groups separately. A further limitation refers to group differences in parts of the outcome measures and predictors, namely, for the athletic burnout dimension physical/ emotional exhaustion as well as for the sport-specific predictor variables athletic identity, excessive effort, and the complementarity subscale of the coach-athlete relationship. However, the results regarding the predictors and moderators cannot be attributed to these group differences, as the analyses were calculated separately for both groups. Finally, the present study only examined general resilience factors calling for the inclusion of more sport-specific and more fine-grained self-concept factors (e.g., athletic self-efficacy and self-esteem) in future studies.

## Conclusion

The present study aimed to achieve two main research objectives. First, we examined predictors of athletic burnout from the three domains of Gustafsson’s integrated model^9^ (major antecedents, entrapment factors, and personality, coping, and environmental factors) along with additional predictors like cognitive styles, in both competitive and leisure athletes. Second, we investigated potential moderators, including performance level and coping resources (ability to bounce back, sense of coherence, self-efficacy, and self-compassion), that could influence the relationship between athletic burnout and life satisfaction. Considering that different performance levels may lead to varying prioritization of sports over other areas of life and potential identity foreclosure with limited experiences outside the sporting context^33^, we compared athletes from different performance levels (competitive vs. leisure sports) for both research objectives.

Indeed, the present study revealed heterogeneous patterns of sport-specific and general predictors of athletic burnout in competitive versus leisure athletes and differentiating moderating factors of the relationship between athletic burnout and life satisfaction in competitive athletes. These differentiating predictors should be considered in prevention or treatment and prevention approaches might benefit from our results by particularly strengthening protective factors to alleviate burnout symptoms and increase life satisfaction in competitive athletes. Furthermore, our results provide starting points for developmental and sport-specific coaching in competitive athletes. For example, personality traits such as excessive effort may be a potential prerequisite for achieving peak performance, while at the same time increasing vulnerability to burnout, a balancing act that can be addressed accordingly in the sports psychology support and personality development of athletes. Further, environmental predictors of burnout, such as coach-athlete relationships, can move more into the focus of support for competitive and elite athletes as they become more relevant during the development of a competitive sport career. This also stresses the need for future longitudinal studies that examine the trajectory and development of athletic burnout together with sport-specific and general protective and vulnerability factors.

## Supplementary Information

Supplementary Information.

## Data Availability

Data will be available in a publicly accessible repository at the latest by the time the paper is published.
