# Supplementary material for: Differences in predicting athletic burnout and in moderating its relationship with life satisfaction in competitive and leisure athletes
Source: Sci Rep. 2024 Oct 22;14:24926. doi: 10.1038/s41598-024-74908-1 (PMC11496630; doi:10.1038/s41598-024-74908-1)
Supplement: Supplementary file 1 — Supplementary Information. [file 41598_2024_74908_MOESM1_ESM.docx]

**Supplemental Material**


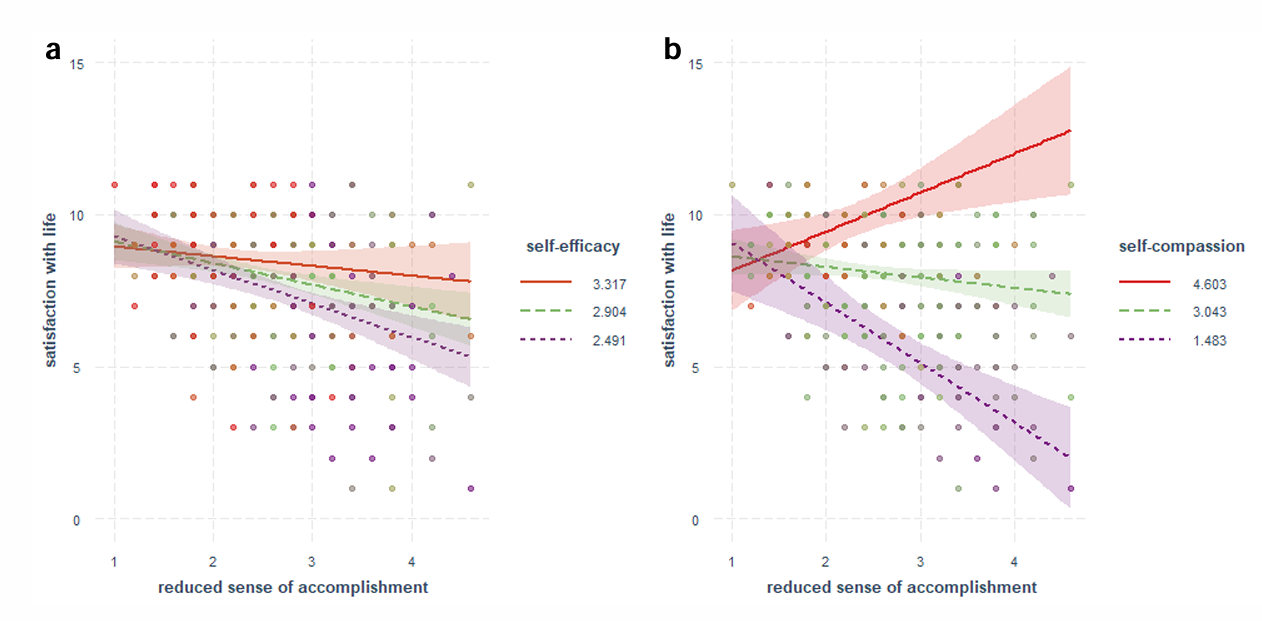


**Figure S1.** Interaction graph for **a** self-efficacy and **b** self-compassion and reduced sense of accomplishment, respectively, on life satisfaction in competitive sports.

**Table S1.**

*Spearman correlations (Holm corrected) of the outcome and predictor variables, separately for competitive sports in the upper range and for leisure sports in the lower range.*

| Variable | 1 | 2 | 3 | 4 | 5 | 6 | 7 | 8 | 9 | 10 | 11 | 12 | 13 | 14 | 15 | 16 | 17 | 18 | 19 | 20 | 21 | 22 | 23 |
| --- | --- | --- | --- | --- | --- | --- | --- | --- | --- | --- | --- | --- | --- | --- | --- | --- | --- | --- | --- | --- | --- | --- | --- |
| 1. AB-physical/ emotional exhaustion |  | .26** | .18 | -.15 | .08 | -.17 | -.08 | -.14 | -.07 | -.24** | .29** | .08 | .08 | .07 | .05 | -.06 | -.09 | .27** | .16 | -.09 | .08 | -.06 | .22* |
| 2. AB-sport devaluation | .22 |  | .44** | -.11 | .09 | -.15 | -.06 | -.16 | -.20* | -.26** | .23** | .03 | .06 | -.34** | -.14 | -.19 | -.24** | .36** | -.02 | -.19 | .03 | -.22* | .11 |
| 3. AB-reduced sense of accomplishment | .11 | .54** |  | -.32** | .30** | -.35** | -.41** | -.40** | -.42** | -.50** | .47** | .25** | .19 | -.15 | -.34** | -.25** | -.31** | .47** | .12 | -.32** | .33** | -.35** | .42** |
| **General predictors** |  |  |  |  |  |  |  |  |  |  |  |  |  |  |  |  |  |  |  |  |  |  |  |
| 4. ability to bounce back | -.27 | -.36* | -.50** |  | -.38** | .31** | .56** | .52** | .33** | .54** | -.49** | -.28** | -.21* | -.04 | -.01 | .02 | .07 | -.22* | -.10 | .09 | -.29** | .08 | -.42** |
| 5. fear of neg. evaluation | -.04 | .14 | .24 | -.36* |  | -.21* | -.45** | -.34** | -.41** | -.41** | .42** | .60** | .53** | .05 | -.05 | -.04 | -.04 | .36** | .30** | -.08 | .55** | -.10 | .42** |
| 6. satisfaction with life | .03 | -.17 | -.41** | .36* | -.31 |  | .45** | .30** | .44** | .64** | -.51** | -.23** | -.09 | .00 | .04 | .09 | .10 | -.24** | -.18 | .12 | -.23** | .12 | -.32** |
| 7. self-compassion | -.14 | -.19 | -.52** | .45** | -.43** | .59** |  | .46** | .50** | .61** | -.52** | -.38** | -.26** | -.05 | .12 | .12 | .12 | -.28** | -.31** | .19 | -.45** | .17 | -.45** |
| 8. self-efficacy | -.04 | -.09 | -.38* | .35* | -.25 | .43** | .40** |  | .47** | .49** | -.36** | -.32** | -.19 | .00 | .12 | .12 | .21* | -.33** | -.04 | .17 | -.20* | .13 | -.39** |
| 9. self-esteem | .14 | -.09 | -.31 | .30 | -.45** | .54** | .48** | .46** |  | .47** | -.37** | -.33** | -.23** | .03 | .14 | .17 | .12 | -.27** | -.05 | .19 | -.24** | .17 | -.32** |
| 10. sense of coherence | -.09 | -.26 | -.50** | .54** | -.35* | .73** | .62** | .54** | .50** |  | -.71** | -.35** | -.24** | .03 | .09 | .14 | .16 | -.40** | -.24** | .18 | -.37** | .20 | -.44** |
| 11. stress | .18 | .29 | .49** | -.55** | .34 | -.63** | -.62** | -.37* | -.50** | -.73** |  | .27** | .22* | .00 | -.06 | -.09 | -.10 | .36** | .19 | -.17 | .37** | -.17 | .49** |
| 12. SC-ability | .01 | .04 | .21 | -.24 | .57** | -.12 | -.21 | -.23 | -.24 | -.26 | .21 |  | .70** | .02 | -.04 | -.04 | -.03 | .39** | .18 | -.11 | .42** | -.07 | .35** |
| 13. SC-opinion | -.04 | .16 | .18 | -.20 | .46** | -.14 | -.16 | -.12 | -.21 | -.21 | .23 | .65** |  | .01 | .00 | .00 | .02 | .33** | .20* | -.07 | .32** | -.03 | .29** |
| **Sport related predictors** |  |  |  |  |  |  |  |  |  |  |  |  |  |  |  |  |  |  |  |  |  |  |  |
| 14. athletic identity | .05 | .03 | -.05 | -.06 | .15 | .07 | -.06 | .23 | .12 | -.01 | .06 | .19 | .15 |  | .08 | .08 | .14 | -.14 | .28** | -.02 | .16 | -.04 | .10 |
| 15. CART-commitment | -.09 | -.16 | -.18 | .11 | .03 | -.12 | -.03 | -.03 | .10 | .06 | .00 | .08 | .13 | .05 |  | .77** | .66** | -.19 | -.12 | .72** | .00 | .71** | -.04 |
| 16. CART-closeness | -.09 | -.09 | -.14 | .06 | .04 | -.03 | .00 | .01 | .01 | .08 | -.02 | .00 | .10 | .03 | .77** |  | .67** | -.13 | -.14 | .69** | .00 | .71** | -.05 |
| 17. CART-complementarity | -.09 | -.14 | -.28 | .05 | .06 | .11 | .18 | .10 | .12 | .24 | -.03 | .09 | .19 | .05 | .73** | .69** |  | -.22* | -.13 | .62** | .03 | .63** | -.08 |
| 18. cognitive interference | .11 | .38* | .50** | -.25 | .11 | -.21 | -.26 | -.30 | -.21 | -.33 | .31 | .17 | .21 | -.18 | -.03 | -.06 | -.19 |  | .14 | -.16 | .32** | -.20 | .40** |
| 19. excessive effort | .12 | .10 | .08 | -.24 | .36* | -.21 | -.32 | -.17 | -.07 | -.30 | .22 | .34 | .23 | .25 | -.07 | -.18 | -.15 | -.01 |  | -.19 | .47** | -.20* | .29** |
| 20. interpersonal satisfaction | .00 | -.23 | -.20 | .11 | -.21 | .00 | .05 | .04 | .11 | .16 | -.05 | -.03 | -.06 | -.12 | .67** | .62** | .63** | -.12 | -.22 |  | -.05 | .64** | -.11 |
| 21. irrational beliefs | .00 | .13 | .36* | -.22 | .57** | -.26 | -.51** | -.15 | -.33 | -.28 | .47** | .40** | .38* | .14 | .01 | .06 | .07 | .20 | .34 | -.16 |  | -.09 | .42** |
| 22. satisfaction with coach and training | -.04 | -.08 | -.16 | .09 | -.01 | -.02 | .09 | -.08 | .16 | .11 | -.05 | .02 | .05 | -.05 | .71** | .68** | .64** | -.02 | -.11 | .64** | -.04 |  | -.12 |
| 23. sport-specific rumination | -.06 | .20 | .37* | -.34 | .37* | -.36* | -.42** | -.29 | -.43** | -.33 | .30 | .23 | .30 | -.01 | .00 | -.05 | -.10 | .30 | .25* | -.15 | .33 | -.19 |  |

*Note.* AB = Athletic burnout, SC = Social comparison, CART = Coach-athlete relationship; * *p* < .05. ** *p* < .01

**Table S2.**

*Validity and reliability of the original version of the English questionnaire used in the study.*

|  | Internal consistency indicated by Cronbach’s α | External validity indicated by significant correlations with following constructs |
| --- | --- | --- |
| Athletic burnout |  |  |
| Physical/ emotional exhaustion | 0.89 | Stress (*r* = .48), amotivation (*r* = .46) |
| Sport devaluation | 0.89 | Stress (*r* = .43), amotivation (*r* = .68) |
| Reduced sense of accomplishment | 0.84 | Stress (*r* = .63), amotivation (*r* = .66) |
| **General predictors** |  |  |
| Ability to bounce back | 0.80-0.91 | CD-RISC (*r* = .59) |
| Fear of negative evaluation | 0.90 | Social avoidance and distress (*r* = .19–.35) |
| Satisfaction with life (single item) | --- | Domain satisfaction (work: *r* = .35; energy: *r* = .42) |
| Self-compassion | 0.87 | Long version of the questionnaire (*r* = .97) |
| Self-efficacy | 0.82-0.93 | Self-esteem (*r* = .52), optimism (*r* = .49) |
| Self-esteem (single item) | --- | Domain-specific self-evaluation (social skills: *r* = .27; physical attractiveness: *r* = .31) |
| Sense of coherence | NA^A^ | NA^A^ |
| Stress | 0.92 | Self-rated stress (*r* = .56), Cohen’s perceived stress scale (*r* = .73) |
| Social comparison orientation | 0.83 | Self-consciousness (*r* = .22–.49), attention to social comparison information (*r* = .47) |
| Ability |  |  |
| Opinion |  |  |
| **Sport related predictors** |  |  |
| Athletic identity | NA^A^ | Task goal orientation (*r* = .26), Win goal orientation (*r* = .34) |
| Coach-athlete relationship |  |  |
| Commitment | 0.83 | Interpersonal satisfaction (*r* = .62) |
| Closeness | 0.86 | Interpersonal satisfaction (*r* = .75) |
| Complementarity | 0.78 | Interpersonal satisfaction (*r* = .59) |
| Cognitive interference | 0.78-0.90 | Subscale “negative thinking” of Test of Performance Strategies |
| Interpersonal satisfaction | 0.83 | Coach athlete relationship (r = .59–.75) |
| Irrational beliefs | 0.76-0.87 | NA^B^ |

*Note.* NA^A^ = not available, as we had no access to the publication, NA^B^ = not available, as no measures on external validity were reported in the paper

**Supplementary references (of the original versions of the questionnaires used):**

1. Raedeke, T. D. & Smith, A. L. Development and Preliminary Validation of an Athlete Burnout Measure. J. Sport Exerc. Psychol. 23, 281–306 (2001).

2. Smith, B. et al. The Brief Resilience Scale: Assessing the Ability to Bounce Back. Int. J. Behav. Med. 15, 194–200 (2008).

3. Leary, M. R. A Brief Version of the Fear of Negative Evaluation Scale. Pers. Soc. Psychol. Bull. 9, 371–375 (1983).

4. Cheung, F. & Lucas, R. E. Assessing the validity of single-item life satisfaction measures: results from three large samples. Qual. Life Res. 23, 2809–2818 (2014)

5. Raes, F.; Pommier, E.; Neff, K.D.; Van Gucht, D. Construction and Factorial Validation of a Short Form of the Self-Compassion Scale. Clin. Psychol. Psychother. 2011, 18, 250–255.

6. Schwarzer, R., & Jerusalem, M. Generalized self-efficacy scale in *Measures in health psychology: A user’s portfolio* (ed. Weinman, J., Wright, S., Johnston, M.). 35–37 (Nfer-Nelson, Windsor, 1995)

7. Robins, R. W., Hendin, H. M. & Trzesniewski, K. H. Measuring Global Self-Esteem: Construct Validation of a Single-Item Measure and the Rosenberg Self-Esteem Scale. Pers. Soc. Psychol. Bull. 27, 151–161 (2001).

8. Antonovsky, A. Unraveling the Mystery of Health: How People Manage Stress and Stay Well. (Jossey-Bass, San Francisco, 1987).

9. Levenstein, S. et al. Development of the perceived stress questionnaire: A new tool for psychosomatic research. J. Psychosom. Res. 37, 19–32 (1993).

10. Gibbons, F. X., & Buunk, B. P. Individual differences in social comparison: Development of a scale of social comparison orientation. *Journal of Personality and Social Psychology*. 76(1), 129–142. [10.1037/0022-3514.76.1.129](https://doi.org/10.1037/0022-3514.76.1.129) (1999).

11. Brewer, B. W., Van Raalte, J. L., & Linder, D. E. (1993). Athletic identity: Hercules’ muscles or Achilles heel? International Journal of Sport Psychology, 24(2), 237–254.

12. Jowett, S. & Ntoumanis, N. The Coach-Athlete Relationship Questionnaire (CART-Q): development and initial validation. Scand. J. Med. Sci. Sports 14, 245–257 (2004).

13. Hatzigeorgiadis, A. & Biddle, S. J. H. Assessing cognitive interference in sport: Development of the thought occurrence questionnaire for sport. Anxiety Stress Coping 13, 65–86 (2000).

14. Turner, M. J. & Allen, M. S. Confirmatory factor analysis of the irrational Performance Beliefs Inventory (iPBI) in a sample of amateur and semi-professional athletes. Psychol. Sport Exerc. 35, 126–130 (2018).
